# STAT3 deficiency in B cells exacerbates uveitis by promoting expansion of pathogenic lymphocytes and suppressing regulatory B cells (Bregs) and Tregs

**DOI:** 10.1038/s41598-020-73093-1

**Published:** 2020-10-01

**Authors:** Favour O. Oladipupo, Cheng-Rong Yu, Ezekiel Olumuyide, Yingyos Jittaysothorn, Jin Kyeong Choi, Charles E. Egwuagu

**Affiliations:** 1 Molecular Immunology Section, Laboratory of Immunology, National Eye Institute (NEI), National Institutes of Health (NIH), Building 10, Room 10N248G, 10 Center Drive, Bethesda, MD 20892-1857 USA; 2 Immunoregulation Section, Laboratory of Immunology, NEI, NIH, Bethesda, MD USA; 3 Department of Immunology, Jeonbuk National University Medical School, Jeonju, Jeonbuk 54896 Republic of Korea

**Keywords:** Inflammation, Neuroimmunology, Immunology

## Abstract

STAT3 transcription factor induces differentiation of naïve T cells into Th17 cells and loss of STAT3 in T cell prevents development of CNS autoimmune diseases. However, function of STAT3 in the B lymphocyte subset is not well understood. In this study, we have generated mice lacking STAT3 in CD19^+^ B cells (CD19-STAT3KO) and investigated intrinsic and extrinsic functions of STAT3 in B cells and its potential role in resistance or pathogenesis of organ-specific autoimmune diseases. We show that STAT3 regulates metabolic mechanisms in B cells with implications for bioenergetic and metabolic pathways that control cellular homeostasis in B cells. Thus, loss of STAT3 in CD19-STAT3KO cells perturbed growth and apoptosis by inducing rapid entry of B cells into the S-phase of the cell cycle, decreasing expression of cyclin-dependent kinase inhibitors and upregulating pro-apoptotic proteins. We further show that the CD19-STAT3KO mice develop severe experimental autoimmune uveitis (EAU), an animal model of human uveitis. Exacerbated uveitis in CD19-STAT3KO mice derived in part from enhanced expression of costimulatory molecules on B cells, marked increase of Th17 responses and increased recruitment of granulocytes into the neuroretina. The enhanced autoimmunity upon deletion of STAT3 in B cells is also recapitulated in experimental autoimmune encephalitis, a mouse model of multiple sclerosis and thus support our conclusion that STAT3 deletion in B cells enhanced inflammation and the effects observed are not model specific. Our data further indicate that STAT3 pathway modulates interactions between B and T cells during EAU resulting in alteration of lymphocyte repertoire by increasing levels of autoreactive pathogenic T cells while suppressing development and/or expansion of immune-suppressive lymphocytes (Bregs and Tregs). Taken together, STAT3 exerts diametrically opposite effects in lymphocytes, with loss of STAT3 in B cells exacerbating uveitis whereas *Stat3* deletion in T cells confers protection.

## Introduction

Signal Transducer and Activator of Transcription 3 (STAT3) regulates critical cellular processes such as, differentiation, cell growth or survival and aberrant activation of STAT3 pathway is implicated in etiology of human and animal diseases^1–4^. STAT3 is an abundant unphosphorylated latent cytoplasmic transcription factor and its biological activity is regulated by post-transcriptional mechanisms that regulate its subcellular localization^5,6^. Prior to activation, the unphosphorylated STAT3 (U-STAT3) exists in the cytoplasm as stable antiparallel dimers, structurally distinct from the activated tyrosine-phosphorylated STAT3 (pSTAT3) dimer^7^. Tyrosine phosphorylation facilitates formation of pSTAT3 dimer that enters the nucleus and activates or represses genes that contain cognate GAS (Gamma-interferon activation site) sequences in their promoter^8^. Although transcription-dependent functions of pSTAT3 are well documented^9^, it is only recently that transcription-independent function of STAT3 in the cytoplasm and mitochondria was discovered^10–14^. Among the non-canonical and non-genomic activities of STAT3 is the capacity of STAT3 to translocate into the mitochondria where it regulates the activity of the electron transport chain^15^. STAT3 activity has broad consequences for cell survival, production of ATP and reactive oxygen species under normal or pathological conditions^16^.

While STAT3 promotes proliferation in most mammalian cell types^1,2^, it plays the unique role of maintaining immune homeostasis by maintaining naïve or resting T cells in the quiescent state (G0 cell cycle phase) and preventing pre-mature T cell proliferation through upregulation of two Forkhead-box (FOX) transcription factors, FOXO1 and FOXO3a^17,18^. STAT3 also influences cell-fate decisions of differentiating naïve T cells, induces differentiation of pathogenic Th17 subset that mediate inflammatory diseases and promotes recruitment of inflammatory cells into sites of inflammation^19^. Importantly, mice with targeted-deletion of STAT3 in the CD4 T cell compartment do not develop experimental autoimmune uveoretinitis (EAU) or experimental autoimmune encephalomyelitis (EAE)^20^. STAT3 mediated resistance to these CNS autoimmune diseases derives from inability to produce Th17 cells and promotion of exaggerated expansion in Foxp3-, IL-10-, IL-4-, and IFN-γ-expressing T cells.

In contrast to the wealth of knowledge on the role of STAT3 in T cells, limited studies have investigated the functions of this critical transcription factor in B cells. However, clinical studies have shown that loss-of-function mutations in DNA-binding domain of STAT3 in B cells contributes to the development of hyper IgE syndrome^21,22^. In the mouse, STAT3 has also been shown to serve as a negative regulator of IgE Class switching and exacerbates lung inflammation^23^. On the other hand, the role of STAT3 in regulating CD19^+^ B-cell lineage commitment, most B cell effector functions, and its role in susceptibility or resistance to autoimmune diseases require further investigations. For example, CNS autoimmune diseases such as multiple sclerosis or uveitis are tacitly viewed as T cell mediated diseases. However, the role of B cells in these diseases is a matter of some debate and needs to be rigorously examined. In this study, we generated mice with targeted deletion of STAT3 in B cells and investigated intrinsic and extrinsic functions of STAT3 in B cell, with particular focus on the role of STAT3 in uveitis, the mouse model of human uveitis.

## Methods

### Mice

Six- to 8-week-old C57BL/6J mice (stock number 000664) and CD19^CRE^ mice (stock number 006785) were purchased from Jackson Laboratory (Jackson Laboratory, Bar Harbor, ME). *Stat3*^fl/fl^ mice were kindly provided by Dr. Davis E. Levy (New York University) as described in our previous paper^20^. CD19-STAT3KO (CD19^+/CRE^STAT3^f/f^) mice were characterized by the tail-DNA PCRs and Western blot analysis of total protein extracts from activated CD19^+^ B cells. The control mouse strain (CD19^CRE/+^STAT3^+/+^) was derived by cross-breeding C57BL/6J with CD19-CRE homozygous mice. Animal care and experimentation conformed to National Institutes of Health (NIH) guidelines. The mice were maintained and treated in accordance with National Eye Institute (NEI) and NIH Animal Care and Use Committee guidelines (Study # EY000262-19 & EY000372-14). The experimental protocol was approved under NIH/NEI Animal Study Protocol (ASP) # NEI-597.

### Experimental autoimmune uveitis (EAU)

EAU was induced by active immunization of C57BL/6J or CD19-STAT3KO mice with IRBP_651-670_-peptide in a 0.2 ml emulsion (1:1 v/v with complete Freund’s adjuvant (CFA) containing *Mycobacterium tuberculosis* strain H37RA (2.5 mg/ml). Mice also received *Bordetella pertussis* toxin (0.2 µg/mouse) concurrently with immunization^24^. For each study, 8–10 mice were used per group and they were matched by age and sex. Clinical disease was established and scored by fundoscopy and histology as described previously^19,25^. Eyes were examined for disease severity using binocular microscope with coaxial illumination. Eyes for histology were enucleated 21 days post-immunization, fixed in 10% buffered formalin and serially sectioned in the vertical pupillary-optic nerve plane. All sections were stained with hematoxylin and eosin.

### Fundoscopy

Funduscopic examinations were performed at day 10 to 21 after EAU induction. Briefly, following administration of anesthesia [intraperitoneal injection of ketamine (1.4 mg/mouse) and xylazine (0.12 mg/mouse)], the pupil was dilated by topical administration of 1% tropicamide ophthalmic solution (Alcon Inc., Fort Worth, Texas). Fundus image was captured using Micron III retinal imaging microscope (Phoenix Research Labs) for small rodent or a modified Karl Storz veterinary otoendoscope coupled with a Nikon D90 digital camera, as previously described^19,26^. To avoid a subjective bias, evaluation of the fundus photographs was conducted without knowledge of the mouse identity by a masked observer. At least 6 images (2 posterior central retinal view, 4 peripheral retinal views) were taken from each eye by positioning the endoscope and viewing from superior, inferior, lateral and medial fields and each individual lesion was identified, mapped and recorded. The clinical grading system for retinal inflammation was as established^27,28^.

### Imaging mouse retina by spectral-domain optical coherence tomography (SD-OCT)

Optical coherence tomography (OCT) is a noninvasive procedure that allows visualization of internal microstructure of various eye structures in living animals. An SD-OCT system with 820 nm center wavelength broadband light source (Bioptigen, NC) was used for in vivo non-contact imaging of eyes from control or EAU mice. Mice were anesthetized and the pupils dilated as described above. Mice were then immobilized using adjustable holder that could be rotated easily allowing for horizontal or vertical scan scanning. Each scan was performed at least twice, with realignment each time. The dimension of the scan (in depth and transverse extent) was adjusted until the optimal signal intensity and contrast was achieved. Retinal thickness was measured from the central retinal area of all images obtained from both horizontal and vertical scans from the same eye, using the system software, and averaged. The method used to determine the retinal thicknesses in the system software was as described^29^.

### Electroretinogram (ERG)

Before the ERG recordings, mice were dark-adapted overnight, and experiments were performed under dim red illumination. Mice were anesthetized with a single intraperitoneal injection of ketamine (1.4 mg/mouse) and xylazine (0.12 mg/mouse) and pupils were dilated with Midrin P containing of 0.5% tropicamide and 0.5% phenylephrine hydrochloride (Santen Pharmaceutical Co., Osaka, Japan). ERGs were recorded using an electroretinography console (Espion E2; Diagnosys LLC, Lowell, MA, USA) that generated and controlled the light stimulus. Dark-adapted ERG was recorded with single-flash delivered in a Ganzfeld dome with intensity of − 4 to 1 log cd s/m^2^ delivered in 7 steps. Light-adapted ERG was obtained with a 10 cd s/m^2^ background, and light stimuli started at 100 cd s/m^2^ in 6 steps. Gonioscopic prism solution (Alcon Labs, Fort Worth, TX, USA) was used to provide good electrical contact and to maintain corneal moisture. A reference electrode (gold wire) was placed in the mouth, and a ground electrode (subcutaneous stainless steel needle) was positioned at the base of the tail. Signals were differentially amplified and digitized at a rate of 1 kHz. Amplitudes of the major ERG components (a- and b-wave) were measured (Espion software; Diagnosys LLC) using automated and manual methods. Immediately after ERG recording, imaging of the fundus was performed as previously described^30^.

### Experimental autoimmune encephalomyelitis (EAE)

EAE was induced by subcutaneous immunization with 200 µg myelin oligodendrocyte glycoprotein peptide 35–55 (MOG_35-55_) (Sigma, ST Louis, MO) in CFA emulsion, containing 2.5 mg/ml of heat killed, pulverized *Mycobacterium tuberculosis* strain H37RA. The mice also received two doses of 0.3 µg *Bordetella pertussis* toxin (Sigma, St. Louis, MO) on day 0, and day 2 post-immunization by intraperitoneal (i.p.) injection in 100 µl of RPMI 1640 medium containing 0.1% normal mouse serum. The control (*cd19*^+/CRE^*stat3*^+/+^) and CD19-STAT3KO (*cd19*^+/CRE^*stat3*^−/−^) (*n* = 6) were euthanized 17 days post-immunization. The mice were monitored and disease severity was assessed daily by a masked observer. Clinical signs of EAE were graded according to the following scale: 0, No clinical symptoms; 1, clumsiness, incontinence or atonic bladder, flaccid tail; 2, mild paraparesis (trouble initiating movement); 3, moderate paraparesis (hind limb weakness); 4, complete front and hind limb paralysis; 5, moribund state^31^. Spinal cord and brain were harvested 17 days post-immunization and infiltrated lymphocytes and other immune cells were isolated by collagenase digestion followed by Percoll-gradient centrifugation and subsequent analysis by FACS and intracellular cytokine staining.

### Lymphocyte proliferation and co-cultures of CD19^+^ B-cells with uveitogenic T-cells

Primary CD19^+^ B-cells (> 96%) were isolated from the spleen/LN of wild type control and CD19-STAT3KO mice by using anti-CD19 magnetic sorting beads (order number: 130-052-201, Miltenyi Biotec Inc. CA). Purified naïve CD19^+^ B-cells were stimulated with LPS (5 µg/ml) or anti-CD40 (5 μg/ml) plus anti-IgM (5 μg/ml) antibodies in 1 × 10^6^ cells/ml for 2–3 days. Then the cultures were pulsed with [^3^H]-thymidine (0.5 μCi/10 μl/well) for 12 additional hours. The incorporated radioactivity was measured by a scintillation counter with cell filters for 96 well plate (Perkin Elmer, Shelton, CT). To evaluate the activity of uveitogenic T-cells, draining LN and splenocytes isolated from EAU mice were stimulated with IRBP peptides (hIRBP_651-670,_ 20 μg/ml) in 2 × 10^6^ cells/ml for 2–3 days in quintuplet cultures. Then, the cultures were pulsed with [^3^H]-thymidine in the last 12 h. For co-cultures of CD19^+^ B-cells with uveitogenic lymphocytes (1:2 ratio) in total 1 × 10^6^ cells/ml, CD19^+^ B-cells isolated from WT and CD19-STAT3KO mice were used to co-culture with wild types of EAU dLN cells that CD19^+^ B-cells have been depleted by anti-CD19-magnetic beads (order number: 130-052-201, Miltenyi Biotec Inc. CA). Data are presented as mean CPM ± S.E.M. of responses of 5 replicate cultures. For CFSE labeling/assay (Molecular Probes, Inc., Eugene, OR), the cells were cultured for 96 h with anti-CD40 (5 µg/ml) plus anti-IgM (5 µg/ml) antibodies. For evaluation of apoptosis, activated mouse CD19^+^ B cells were washed twice in PBS with 10% FBS. Cells were stained with Annexin V apoptosis detection kit I (BD Biosciences, San Diego, CA, USA), according to manufacturer’s instructions.

### Quantitative and semi-quantitative RT PCR analyses

Total RNA was extracted by using TriZol reagent. All RNA samples were digested with RNase-free DNase I. The quality and quantity of RNA were verified by analysis of 18S and 28S ribosomal RNA expression using Agilent 2100 Bioanalyzer system and Agilent RNA 600 Nano Reagent Kit (Agilent Technologies, Santa Clara, CA). The cDNA synthesis was performed by using superscript III reverse transcriptase and oligo(dT)_12–16_ as recommended by the manufacturer (Invitrogen-Thomas Scientific, Carlsbad, CA). cDNA preparations were normalized to β-actin. Real-time PCR (TaqMan) primers, probes, and reaction buffers (TaqMan fast universal PCR kit 2X) were purchased from Applied Biosystems and TaqMan PCRs were performed in ABI QuantStudio 7 Flex (Applied Biosystems-Thomas Scientific, Carlsbad, CA) as previously described^32^. Taqman probe and primer sets were purchased from Applied Biosystems-Thomas Scientific and the catalogue numbers are listed below: Beta-Actin (Mm02619580_g1), Foxo1 (Mm00490671_m1), Foxo3 (Mm01185722_m1), p2^cip1^ (Mm04207341_m1), p27^kip1^(Mm00438168),Bim (Bcl2L11, Mm00437796_m1), Bax (Mm00432051_m1), Bad (Mm00432042_m1), IL-6 (Mn00446190_m1) IL-1b (Mm00434228_m1), IL-10 (Mm01288386_m1), and TGF-b1(Mm01178820_m1).

### Isolation of PBMC, draining lymph-node, and spleen cells

Control and CD19-STAT3KO EAU mice were euthanized with CO_2_ in accordance to NIH animal user guidelines. Then, their hearts were immediately punched with 1 ml syringe and 25-G needle containing 100 USP units Heparin Sodium (NDC 0069-0062-02, Pfizer Labs, Pfizer Inc, NY). The blood was transferred to a 15 ml tube containing 5 ml of 1 × PBS and then mixed well. 2 ml of Isolymph (Gallard-Schlesinger Industries, Inc. Norway) was carefully added into the bottom of tube to form its under-layer. The sample tubes were centrifuged in 1800 rpm at room temperature 30 min. The whitish inter-phase (containing PBMC) was pulled out around 1 ml and transferred into new 15 ml tube with 9 ml of PBS. The tubes were centrifuged in 1200 rpm at 4 ºC for 7 min. The pellets were washed in 10 ml cold RPMI 1640 medium two times. Finally, PBMCs were suspended into complete RPMI 1640 medium containing 10% FBS (Hyclone, Utah). Draining LN and spleens of control and EAU mice were dissected and were teased with cell strainer (2 mice per the strainer). The cells were suspended in 50 ml of IPMI 1640 medium. After washed for two times, the pellets were suspended into 4 ml of ACK lysing buffer (Quality Biological, MD) for 3–4 min with a vortex per 30 s. Then, 10 times more of RPMI 1640 medium were added into the tubes. After washing for two times, the cells were suspended into completed medium (with 10% FBS, Hyclone, Logan, Utah). The isolated cells were counted in Vi-Cell XR (Viability analyzer, Beckman Coulter, Indianapolis, IN). The cells were immediately used for phenotype identification. For intracellular cytokine staining, the cells were reactivated with PMA (20 ng/ml) and Ionomycin (1 μm) for 5 h and Golgistop as recommended (BD Pharmingen) and was added for last 1 h of the 5 h. Remove spleens and lymph nodes from sacrificed mice^33^.

### CD19^+^ B cell isolation and fluorescence-activated cell sorting (FACS) analysis

Primary mouse CD19^+^ B cells were isolated from PBMC, spleen or draining lymph-node (dLN) using CD19-micro beads (order number: 130-052-201,Miltenyi Biotec Inc. CA). Cells were directly used for surface and intracellular FACS analysis or reactivated with IRBP or anti-CD40/anti-IgM antibodies as previously described^34,35^. For intracellular cytokine detection, sorted CD19^+^ B cells, PBMC, total dLN or splenocytes were re-stimulated for 5 h with PMA (20 ng/ml) and ionomycin (1 μM). GolgiStop (BD Pharmingen, San Diego, CA) was added in the last hour. The intracellular cytokine staining assays were performed using the BD Biosciences Cytofix/Cytoperm kit as recommended by manufacturer (BD Pharmingen, San Diego, CA). FACS analysis was performed on a MACSQuant analyzer (Miltenyi Biotec, San Diego, CA) using labelled monoclonal antibodies and corresponding isotype control Abs (Pharmingen, San Diego, CA). Dead cells were stained with dead cell exclusion dye (Fixable Viability Dye eFluor 450, eBioscience), and live cells were subjected to side-scatter (SSC) and forward-scatter (FSC) analyses. FACS analysis was performed on single cells. Quadrant gates were set using isotype controls with less than 0.5% background.

### Western blotting analysis

Whole cell lysates were prepared as previously described^36^. Protein extracts (20 µg/lane) were fractionated on 4–12% gradient NuPAGE Bis–Tris gels (Invitrogen, Carlsbad, CA) under reduced condition. Blots were probed with anti-STAT3 (Cell Signaling Technology, Danvers MA) or β-Actin (Santa Cruz Biotechnology, Santa Cruz, CA). Western blot assays were performed in Li-Cor two color system on the same membrane.

### The enzyme-linked immunosorbent assay (ELISA)

CD19^+^ B cells, dLN or spleen cells were reactivated in vitro and culture supernatants were collected after a 48-h incubation. Cytokine secretion was quantified using ELISA Kit (R&D Systems, Minneapolis, MN).

### DNA content/cell cycle determination via flow cytometry

Freshly isolated CD19^+^ B cells were stimulated with LPS for 72 h, fixed and stained with propidium iodide. Cells were analyzed at various phases of the cell cycles using FlowJo. Note: cells were treated with RNAse prior to flow cytometry.

### Glycolytic rate assay

Isolated CD19^+^ B-cells from splenocytes were stimulated with anti-IgM and anti-CD40 antibodies (each 5 μg/ml) or LPS (5 μg/ml) for 48 h. Glycolytic rate assays were performed using the Seahorse Glycolytic Rate XFp kit (Agilent Technology, Cedar Creek, TX). Results are presented as Proton Production Rate (PPR).

### Statistical analysis

Statistical analysis was performed by Student *t*-test (two-tailed). Asterisks denote p value (*P < 0.05, **P < 0.01, ***P < 0.001, ****P < 0.0001).

## Results

### Generation and characterization of STAT3 conditional KO mice

**Figure 1:**
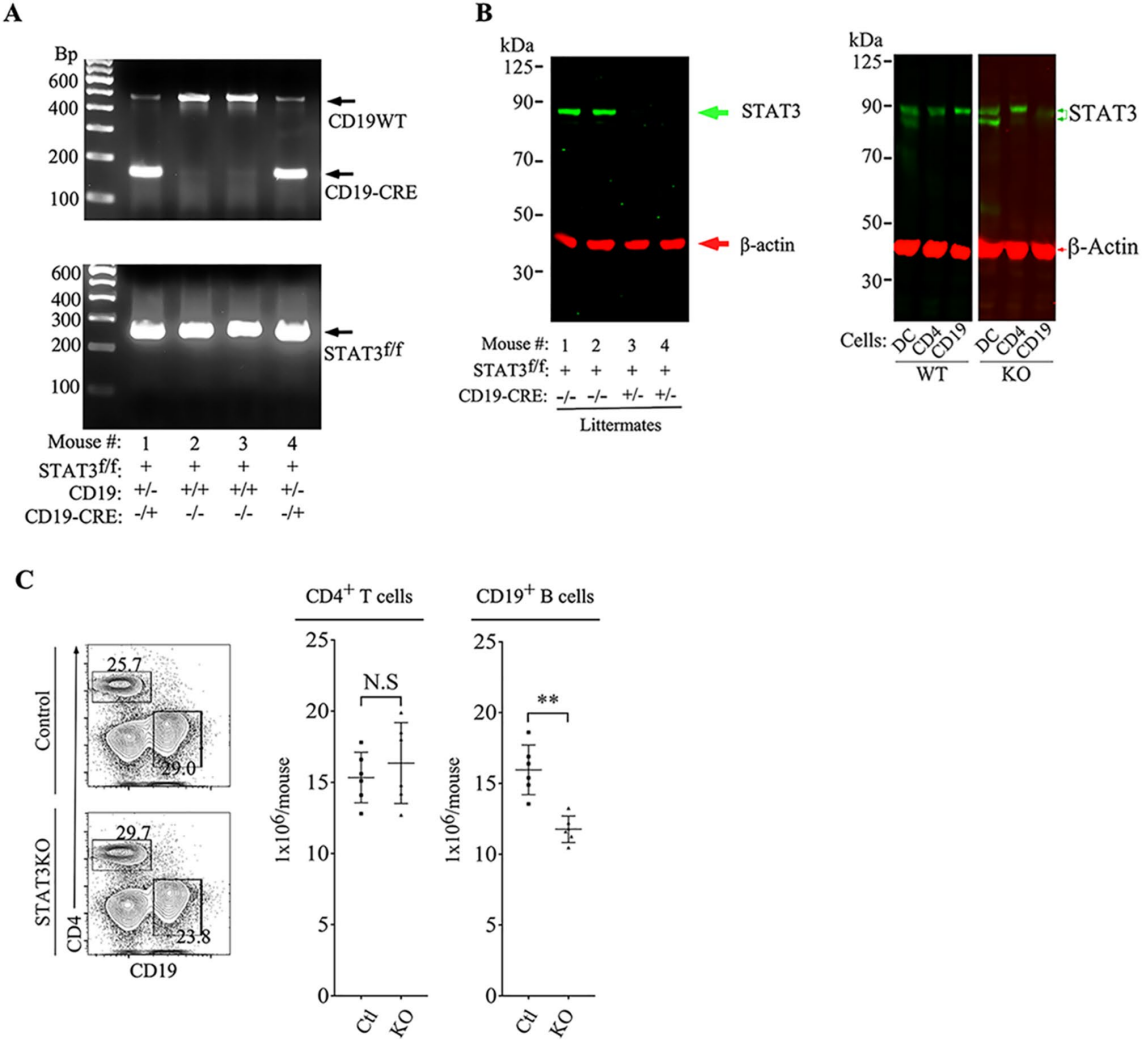
Generation and characterization of STAT3 conditional KO mice. *Stat*3^fl/fl^ mice were crossed with CD19-Cre mice to generate mice with deletion of *stat3* in CD19^+^ B cells (CD19-STAT3KO). (**A**) CD19-STAT3KO mice were identified by PCR analysis of mouse tail genomic DNA. (**B**) Naive CD19^+^ B cells from LN and spleens of control (CD19^+/CRE^STAT3^f/f^) or CD19-STAT3KO mice were stimulated with LPS and analyzed by Western blotting. (**C**) Splenocytes were isolated from control or CD19-STAT3KO mice and cells were counted and immediately used for surface FACS analysis with anti-CD4 or anti-CD19 mAbs after dead cell exclusion. Numbers in the quadrants are the percentage of CD4^+^ T cells or CD19^+^ B cells. Histograms presented in (**C**) represent the absolute number of T cells or B cells per mouse (n = 6). Results represent at least 3 independent studies. *p < 0.05, **p < 0.01, ***p < 0.001, N.S (Student two-tailed t test).

We generated mice with targeted deletion of *Stat3* in CD19^+^ B cells (CD19-STAT3KO) to investigate intrinsic and extrinsic functions of STAT3 pathway in B cell development and during central nervous system (CNS) autoimmune disease. PCR analysis of tail DNA of mice from the cross between CD19-Cre and *Stat3*^fl/fl^ mouse strain (C57BL/6J background) confirmed the generation of CD19-Cre/STAT3^fl/fl^ double-positive mice (Fig. 1A). The strain was maintained as *cd19*^+/−^*stat3*^−/−^ (CD19-STAT3KO) and crossed with *stat3*^fl/fl^ mice to generate the CD19-STAT3KO used in all experiments described herein. Use of the mouse strain expressing Cre recombinase under the CD19 promoter element (CD19-Cre strain) resulted in targeted deletion of *Stat3* in CD19^+^ B cells. Protein extracts derived from sorted CD19^+^ B cells in the lymph nodes (LN) and spleen of control or CD19-STAT3KO mice were analyzed by Western blot and results show that the STAT3 protein was not detectable (Fig. 1B), confirming that *stat3* was indeed deleted in the CD19-STAT3KO B cells. To confirm that effects of the loss of STAT3 is restricted to B cells and does not extend to other immune cell types, we analyzed dendritic cells (DC), CD4^+^ T cells or CD19^+^ B cells from WT or CD19-STAT3KO mice and confirmed by Western blotting that STAT3 is expressed by DC or T cells from the CD19-STAT3KO cells but not the B cells (Fig. 1B, right panel).

We also determined the absolute numbers of CD4^+^ T cells and CD19^+^ B cells in the spleen of control (CD19^+/CRE^STAT3^+/+^) or CD19-STAT3KO mice. The numbers of T cells were unaffected while we observed significant diminution of CD19^+^ B cells (Fig. 1C), further indicating that effects of the loss of STAT3 is restricted to the B cell compartment. The CD19-STAT3KO mouse is characterized by IgM^hi^CD21^hi^CD23^hi^CD24^hi^CD1d^hi^ B cell immunophenotype (Supplementary Figure 1), suggesting defect in making the transition from T-2-transitional to mature CD19-STAT3KO B cells^37^. These results also suggest that STAT3 pathway might be required for regulating maturation and homeostatic expansion of CD19^+^ B-cells and may have a unique role of limiting the in vivo levels of marginal zone B cells (MZ).

### STAT3 regulates B cell activation and proliferation

**Figure 2:**
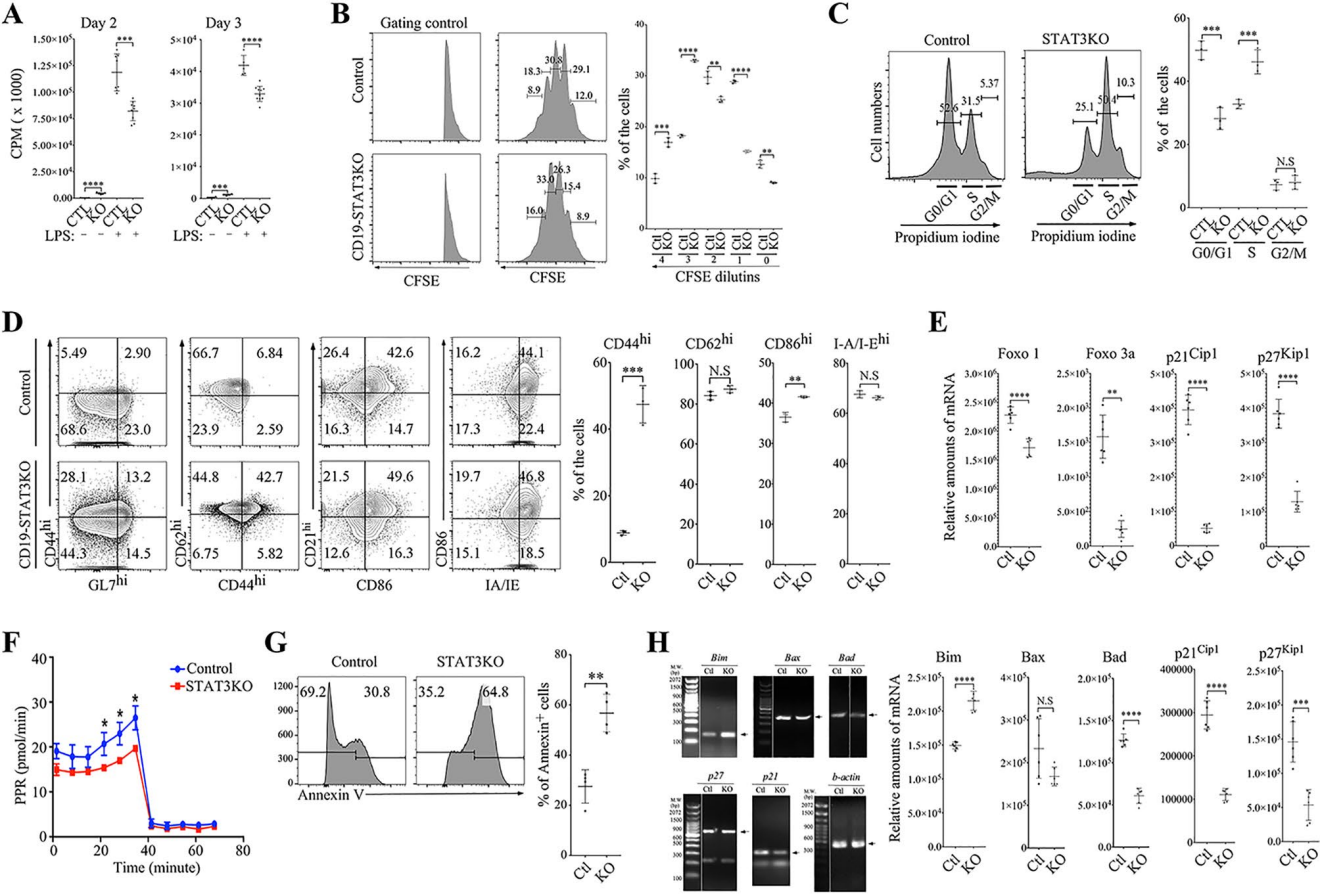
STAT3 regulates B cell proliferation and STAT3-deficient B cells exhibit a pre-activated phenotype. (**A**–**C**) CD19^+^ B cells isolated from control or CD19-STAT3KO mouse spleen were activated with LPS and subjected to thymidine incorporation assay (**A**), CFSE dilution assay (**B**) or DNA analysis by the propidium iodine assay (**C**). (**D**) Freshly isolated B cells from the spleen were stained with Abs specific to cell surface proteins indicated and analyzed by FACS. Graphs show relative amounts of the CD19^+^ B cells expressing the various cell surface markers (mean and SEM, n = 6). (**E**) Naive CD19^+^ B cells from spleen of control and CD19-STAT3KO mice were analyzed by qPCR. (**F**) Purified CD19^+^ B cells from naïve control or CD19-STAT3KO mouse spleen were activated with anti-CD40 and anti-IgM antibody for 48 h and glycolic assays were performed using the Seahorse Glycolytic Rate XFp kit (Agilent Technology, Cedar Creek, TX). Results are presented as Proton Production Rate (PPR). (**G**) Apoptosis assays were performed using CD19-STAT3KO or control B cells activated with anti-CD40 and anti-IgM antibodies for 48 h. (**H**) RT-PCR analysis of RNA obtained from the activated B cells and bands detected by gel electrophoresis were quantified by qPCR (right panels). Results represent at least 3 independent studies. *p < 0.05, **p < 0.01, ***p < 0.001, N.S (Student two-tailed t test).

Several reports have shown that STAT3 controls growth and differentiation of many cell types including lymphocytes^1,2,17,18^. To investigate the role of STAT3 in B cell activation and proliferation, we sorted B cells from WT and CD19-STAT3KO mice and stimulated the cells with LPS for 72 h. Analysis of the cells by the thymidine incorporation revealed significant decrease in proliferative responses by the CD19-STAT3KO compared to WT B cells (Fig. 2A). This result suggests that STAT3 functions to promote B cell proliferation and might therefore be a therapeutic target for regulating B cell mediated autoimmunity. However, cell division measured by CFSE dilution analysis of labeled live cells revealed a complex role of STAT3 in regulation of B cell proliferation. At earlier stages of the cell cycle, WT B cells proliferated faster but after the third cycle of proliferation we observed a dramatic switch indicated by a faster cell division rate by the STAT3-deficient B cells compared to wild type controls (Fig. 2B). This observation is consistent with results of DNA content cell cycle analysis showing higher percentage of WT B cells at G0/G1 phase while STAT3-deficient B cells were significantly higher at the S-phase (Fig. 2C). In addition, CD19-STAT3KO B cells exhibited an enhanced activation phenotype, as indicated by increased proportion of cells expressing CD44^+^GL7^+^ activated B cells (Fig. 2D). Analysis of RNA from naive CD19^+^ B cells from spleen of control and CD19-STAT3KO mice by qPCR revealed reduced transcription of genes that code for lymphocyte quiescence transcription factors (Foxo1 and Foxo3a) and cyclin-dependent kinase inhibitors (p21^Cip1^ and p27^Kip1^), indicating that the CD19-STAT3 T cells are poised for activation (Fig. 2E). Enhanced activation and proliferation of immune cells is generally associated with increased glycolysis. We therefore activated CD19^+^ B cells from control or CD19-STAT3KO mice for 72 h and assessed whether the increase in proliferative response of CD19-STAT3KO would correlate with increase in glycolytic activity. Surprisingly, analysis of their glycolytic activities revealed significantly lower glycolytic rate in the CD19-STAT3KO B cells (Fig. 2G). This result is consistent with the thymidine incorporation assay, suggesting decreased numbers of cells in the cultures after 3 days stimulation with LPS. To examine whether the enhanced proliferation at the S-phase of the cell cycle might render the CD19-STAT3KO B cells highly susceptible to apoptosis, we analyzed WT and CD19-STAT3KO B cells by the Annexin V staining assay and found twofolds higher level of CD19-STAT3KO undergoing apoptosis compared to control B cells (Fig. 2G) and this result correlated with increased expression of Bim-1 by CD19-STAT3KO B cells (Fig. 2H).

### CD19-STAT3KO mice developed severe experimental autoimmune uveitis (EAU)

**Figure 3:**
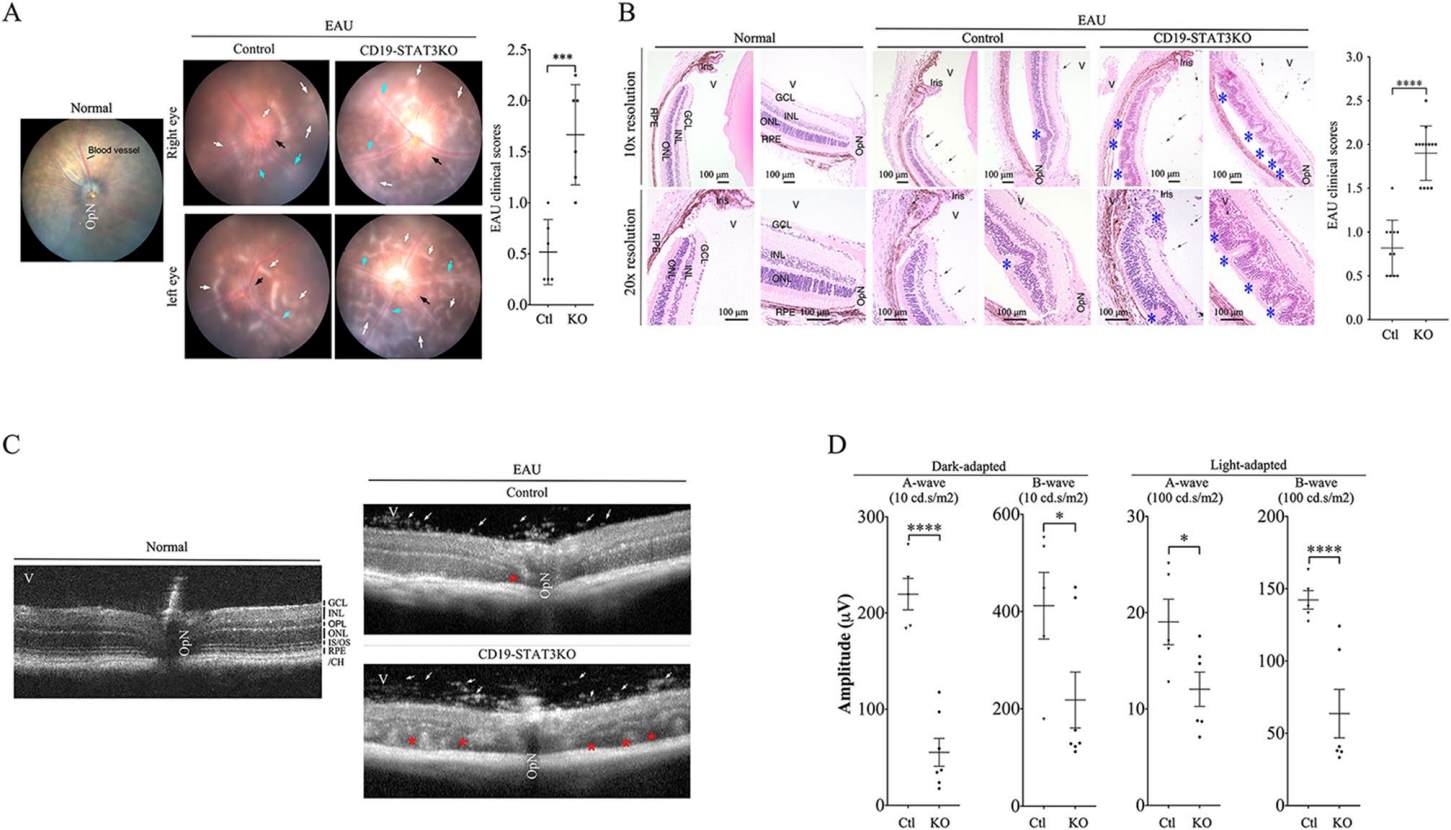
CD19-STAT3KO mice developed severe EAU. EAU was induced in control or CD19-STAT3KO mice by immunization with the uveitogenic peptide, IRBP_651-670_ in CFA and disease progression was analyzed by fundoscopy, histology, optical coherence tomography (OCT) and electroretinography (ERG). (**A**) Fundus image of retina at day 21 after EAU induction were taken using an otoendoscopic imaging system. Fundus images reveal inflammation with blurred optic disc margins and enlarged juxtapupillary area (black arrows), retinal vasculitis (blue arrows) and yellow-whitish retinal and choroidal infiltrates (white arrows). OpN, optic nerve. Clinical scores and assessment of disease severity were based on changes at the optic nerve disc or retinal vessels and retinal and choroidal infiltrates. (**B**) Histologic images of the eyes were from eyes harvested 21 days after EAU immunization and show substantial numbers of inflammatory cells in the CD19-STAT3 retina. H&E histological sections: Scale bar, 100 µM. V, vitreous; GCL, ganglion cell layer; INL, inner nuclear layer; ONL, outer nuclear layer; RPE/CH retinal pigmented epithelial and choroid. Blue arrows, lymphocytes; Asterisks, retinal-folds. Histogram to the right shows the clinical scores, N = 10. (**C**) Representative OCT images show marked increase of inflammatory cells (white arrows) in the vitreous of mice and disturbance of the retinal layer structure with granulomatous lesions (red arrows) were more pronounced in the CD19-STAT3KO retina. (**D**) ERG analysis of the retina on day-20 after EAU induction. The averages of light- or dark-adapted ERG a-wave or b-wave amplitudes are plotted as a function of flash luminance and values are means ± SEM from 4 animals in each group. Data are presented as the mean ± SEM of at least three determinations. Results represent 3 independent studies. **p < 0.01, ***p < 0.001, ****p < 0.0001.

To investigate the impact of deleting STAT3 in CD19^+^ B cells on the development and severity of uveitis, we induced EAU in WT control and CD19-STAT3KO mice by active immunization with the uveitogenic peptide, IRBP_651-670_ in CFA emulsion^24^. Initial signs of uveitis in the C57BL/6J EAU model are generally observed by day 12 post-immunization (p.i.), with full-blown uveitis occurring between day 16 and day 22 p.i. Disease progression was monitored by fundoscopy, histology, optical coherence tomography (OCT) and electroretinography (ERG). Fundoscopic images obtained on day 21 p.i showed the development of ocular inflammation in the WT mouse eyes characterized by blurred optic disc margins and enlarged juxtapapillary areas, retinal vasculitis with moderate cuffing, vitreitis, choroiditis and yellow-whitish retinal and choroidal infiltrates (Fig. 3A). In contrast, the fundus images reveal more severe disease in the eyes of CD19-STAT3KO mice with significantly higher clinical scores compared to the eyes of WT mice (Fig. 3A). Histological analysis of retinas from eyes harvested 21 days p.i underscored the severity of EAU in the eyes of CD19-STAT3KO mice. Compared to EAU in the WT mice, disease in the CD19-STAT3KO mice was characterized by the infiltration of large numbers of inflammatory cells into the retina resulting in substantial destruction of retinal cells, development of more retinal folding, serous retinal detachment, vasculitis, retinitis, choroiditis, and vitreitis (Fig. 3B). Optical coherence tomography is a noninvasive procedure that allows visualization of internal microstructure of various eye structures in living animals. OCT analysis revealed substantial accumulation of inflammatory cells in vitreous and optic nerve head of CD19-STAT3KO eyes compared to WT eyes (Fig. 3C). Inflammation of the retina induces changes in the electroretinogram (ERG) which measures changes in electrical potentials in response to light stimulation of the retina and is a well-established tool for identifying gross physiologic changes indicative of alterations in visual function^19,38^. in the intact retina. The two major ERG waves are the photoreceptor-derived a-wave and the b-wave that derives from bipolar cells in the inner nuclear layer (INL). ERG under light-adaptive stimuli reflects cone-driven signaling, while the dark-adapted b-wave responses represent mainly the rod-driven signaling. Analysis of light-adapted or dark-adapted ERG on day 20 postimmunization showed significantly lower a- and b-wave amplitudes in eyes of CD19-STAT3KO mice (Fig. 3D). This suggests significant decline of visual impairment attributable to defects in cone and rod signaling functions and is consistent with higher clinical pathological score in CD19-STAT3KO EAU mice (Fig. 3A,B). Across the board all hallmarks of severe uveitis were observed in CD19-STAT3KO mice.

### STAT3 deficiency in B cells promotes expansion of pathogenic Th17 cells during EAU

**Figure 4:**
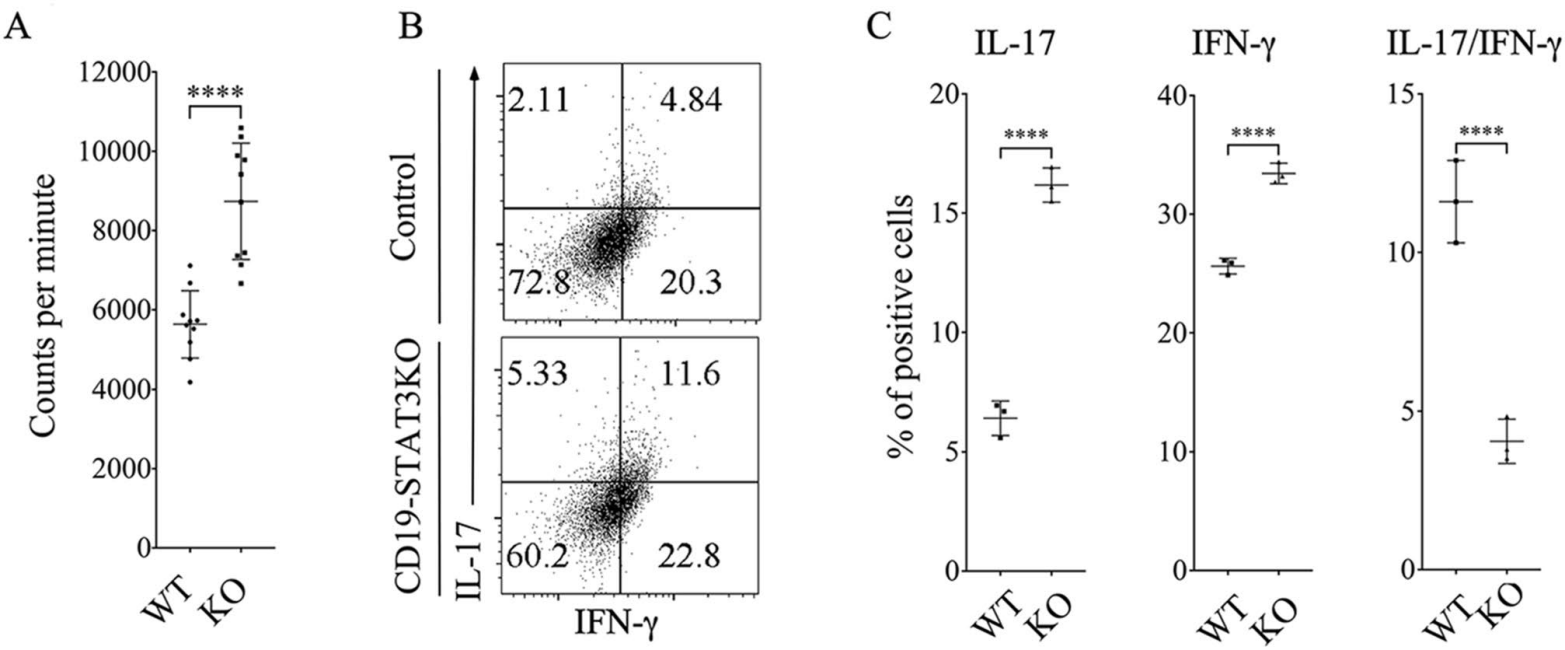
Exacerbated EAU in CD19-STAT3KO mice correlate with expansion of Th17 cells. (**A**) Lymphocytes were isolated from control or CD19-STAT3KO EAU mice and proliferative responses of the cells to IRBP_651-670_ were assessed by ^3^H-thymidine incorporation assay. Five replicate cultures were analyzed, and data presented as mean value of CPM of the five replicate cultures. (**B**,**C**) PBMC from peripheral blood of control or CD19-STAT3KO EAU mice were stained and analyzed by intracellular cytokine staining assay and FACS. Numbers in quadrants indicate percentage of T cells expressing IL-17 and/or IFN-γ. Data are presented as mean ± SEM of three replicates. Results represent 3 independent studies. **p < 0.01, ***p < 0.001, ****p < 0.0001.

EAU is a T cell mediated intraocular inflammatory disease and IL-17 and/or IFN-γ-producing T cells are implicated in its etiology^39^. We therefore investigated whether the development of severe uveitis in CD19-STAT3KO mice derived from aberrant expansion of IRBP-specific pathogenic T cells and inflammatory responses induced by Th17 and/or Th1 cells. We isolated lymphocytes from EAU mice, re-stimulated the cells with IRBP_651-670_ and ^3^H-thymidine incorporation assay showed that CD19-STAT3KO T cells exhibited higher proliferative responses compared to WT T cells (Fig. 4A). Analysis of the cells by intracellular cytokine staining showed increase of IL-17-producing Th17 cells in the CD19-STAT3KO mice (Fig. 4B,C). The expansion of Th17 cells expressing both IL-17 and IFN-γ (DP-Th17) is of noteworthy as the DP-Th17 population is implicated in severe organ-specific autoimmune diseases^39,40^.

### Loss of STAT3 in B cells antagonizes expansion of regulatory T and B cells

**Figure 5:**
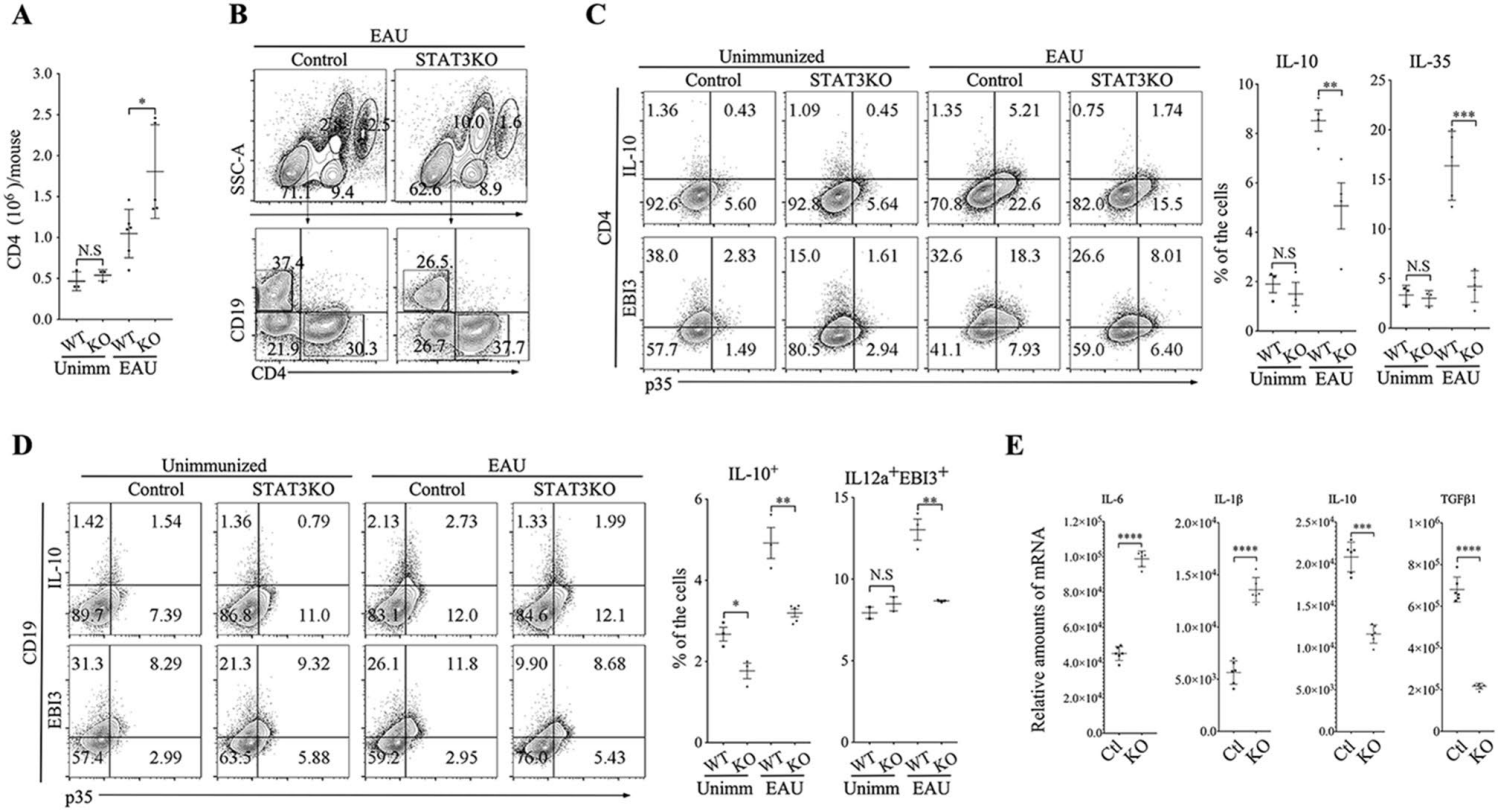
CD19-STAT3KO mice are defective in generating Breg and Treg cells during EAU. (**A**) Freshly isolated CD4^+^ T cells in the dLNs and spleen of EAU mice were re-stimulated with IRBP and counted using the Vi-Cell XR cell viability analyzer (Beckman Coulter). (**B**) Freshly isolated PBMC cells from control or CD19-STAT3KO EAU mice were gated by FACS for CD11b^+^, CD4^+^, and CD19^+^ cells after dead cell exclusion. (**C**) CD4^+^ T cells or (**D**) CD19^+^ B cells expressing IL-10 or IL-35 (p35 and EBI3) were detected and quantified by the intracellular cytokine staining assays. Numbers in quadrants and the corresponding histograms indicate percentage of IL-10-, p35- and/or Ebi3-expressing cells. (**E**) RNA isolated from the CD19^+^ B cells were analyzed by qPCR. The data are presented as the mean ± SEM of at least three determinations. *p < 0.05, **p < 0.01, ***p < 0.001, ****p < 0.0001.

Regulatory B cells (Bregs) and regulatory T cells (Tregs) that suppress pro-inflammatory T cells and autoimmune responses require STAT3 pathways to mediate their inhibitory functions^25,41,42^. Here, we tested whether severe EAU in CD19-STAT3KO mice derived in part from increase in pathogenic T cells during EAU and corresponding defect in generating Breg and/or Treg cells that suppress unbridled proliferation of uveitogenic T cells in the retina. First, we isolated CD4^+^ T cells in the dLNs and spleen of EAU mice, re-stimulated with IRBP and consistent with increased proliferation of CD19-STAT3KO T cells, the absolute numbers of T cells was significantly higher in EAU CD19-STAT3KO compared to WT mice (Fig. 5A). We also gated for CD4^+^ or CD19^+^ lymphocytes and as shown by FACS analysis the CD19-STAT3KO mice higher percentage of CD4^+^ T cells compared to WT (37.7% versus 30.3%) while percentage of CD19^+^ B cells in the WT was higher (37.4%) compared to 26.5% B cells in CD19-STAT3KO mice (Fig. 5B). Intracellular cytokine analysis performed on the gated CD4^+^ population revealed expansion of IL-10 producing T cells (Tregs) and IL-35-producing Tregs (Fig. 5C). On the other hand, these regulatory Treg populations were markedly reduced in the CD19-STAT3KO mice with EAU (Fig. 5C). Analysis of gated CD19^+^ B cells showed marked reduction of IL-10 producing Bregs and IL-35-producing Bregs (Fig. 5D). Interestingly, we observed significant reduction of IL-10-producing Tregs and IL-35-producing Tregs in the blood, dLNs and spleen. However, while Foxp3^+^ Treg cells were reduced in the blood, we observed equivalent levels of Foxp3^+^IL-10^+^ or Foxp3^+^IL-35^+^ Tregs in the spleen and dLNs suggesting that all Treg subtypes were inhibited in the CD19-STAT3KO mice during EAU (Supplementary Fig. 3). These results are consistent with qPCR analysis showing downregulated transcription of the immune suppressive genes (*il10* gene and *tgfβ*) and upregulation of proinflammatory genes (*il6* and *il1β*) of CD19-STAT3KO B cells (Fig. 5E) and suggest that loss of STAT3 in B cells antagonizes expansion of regulatory T and B cells.

### CD19-STAT3KO mice upregulate B cell co-stimulatory and inhibitory receptor molecules

**Figure 6:**
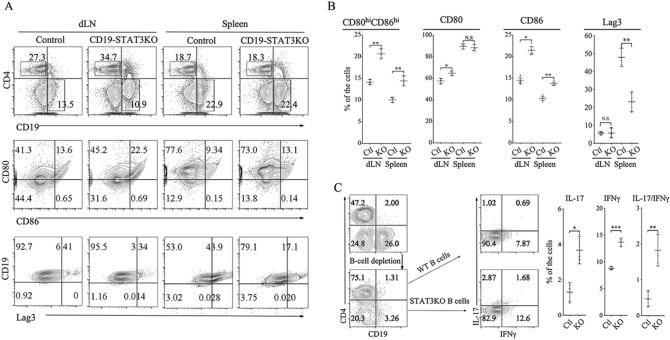
Upregulation of CD80 anf CD86 and downregulation of Lag3 on CD19-STAT3KO B cells during EAU. **A** Draining LN and splenocytes from control or CD19-STAT3KO EAU mice were gated by FACS analysis into CD4^+^, CD19^+^ cells after dead cell exclusion. **B** Numbers in quadrants **A** and corresponding histograms indicate percentage of CD19^+^ cells expressing costimulatory molecules (CD80, CD86) or LAG3. (**C**) CD19^+^ B-cells from control or CD19-STAT3KO EAU mice were co-cultured with B cell-depleted dLN cells from control EAU mice (1:1) for 3-days in medium containing IRBP_651-670_. Intracellular cytokine analysis was performed and numbers in quadrants and histograms indicate percentages of IL-17- and IFN-γ-expressing CD4^+^ T cells. The data are presented as the mean ± SEM of at least three determinations. *p < 0.05, **p < 0.01, ***p < 0.001, ****p < 0.0001.

CD80 (B7-1), and CD86 (B7-2) are costimulatory molecules expressed on antigen-presenting cells including dendritic cells, macrophages and activated B cells. Expression of CD80 and CD86 costimulatory molecules rapidly increase following activation of CD19^+^ B-cells in response to B cell receptor (BCR) or TLR agonist. We isolated lymphocytes from the dLN and splenocytes of EAU mice and used equivalent numbers of WT and CD19-STAT3KO B cells for analysis of cells expressing CD80 or CD86. Both CD80 and CD86 were upregulated in the CD19-STAT3KO compared to WT B cells, indicating that loss of STAT3 in B cells correlates with increase in molecules associated with B cell-mediated antigen presentation (Fig. 6A). These results thus suggest that in B cells, STAT3 may serve to attenuate expression of costimulatory molecules and thereby restrains excessive activation of T cells. Lag3 is an inhibitory receptor that induces T cell exhaustion and suppresses excessive T cell responses that might induce autoimmunity. Consistent with expansion of uveitogenic CD19-STAT3KO B cells, Lag3 expression is reduced on CD19-STAT3KO B cells (Fig. 6A,B). Increased expression of co-stimulatory molecules on CD19-STAT3KO B cells might enhance Ag presentation to uveitogenic T cells and activation of T cells in CNS tissues is thought to perpetuate neuroinflammation. We therefore examined whether CD19^+^ B cells from CD19-STAT3KO EAU mice might exerted direct effects on the expansion of the IRBP-specific uveitogenic T cells. Analysis of CD4^+^ T cells at day 7 post-immunization showed significant proliferation of the CD19-STAT3KO T cells compared to WT, providing suggestive evidence that STAT3KO B cells might enhance Ag presentation to uveitogenic T cells (Supplementary Figure 2). To directly examine effect of the loss of STAT3 in B cells on expansion of proinflammatory uveitogenic T cells we first isolated CD19^+^ B cells from the draining LN (dLN) and spleen of WT or CD19-STAT3KO with EAU mice. We next isolated cells from the dLN of WT EAU mice, depleted them of CD19^+^ B cells using CD19^+^ magnetic beads and co-culture the B cell-depleted dLN cells with the WT or CD19-STAT3KO B cells (1:1 ratio) for 3 days in medium containing IRBP_651-670_. Intracellular cytokine staining assay showed that the CD4 T cells produce more inflammatory cytokines when co-cultured with CD19-STAT3KO B cells (Fig. 6C), providing suggestive evidence that CD19-STAT3KO B cells might have direct effects on the activation and expansion of uveitogenic T cells.

### CD19-STAT3KO mice develop severe encephalomyelitis

**Figure 7:**
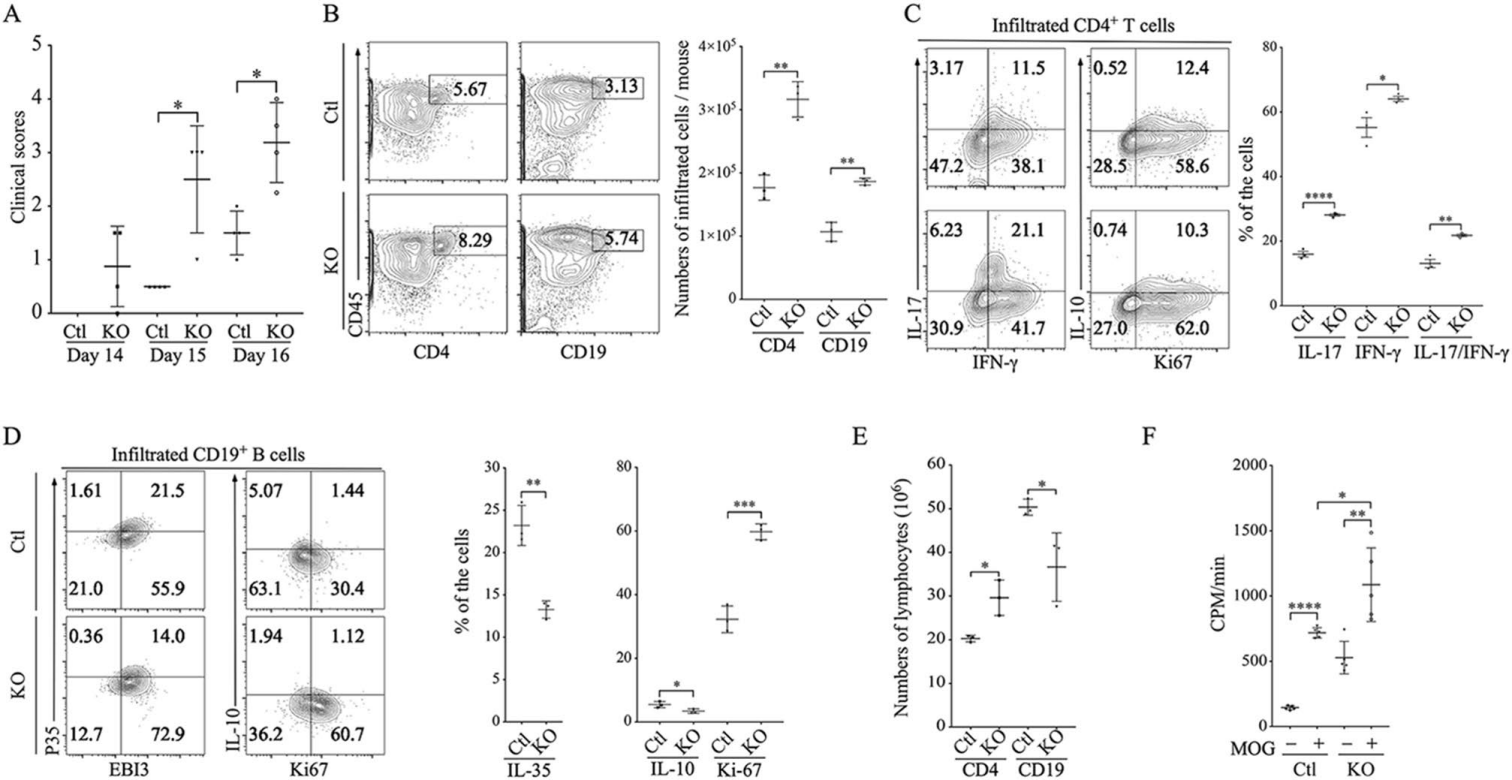
CD19-STAT3KO mice developed severe EAE. (**A**) EAE was induced in CD19-STAT3KO (N = 4) or WT control mice (N = 4) by immunization with MOG_35-55_-peptide in CFA. Clinical disease scores were assessed daily, from day 8 until day 16, by masked investigators. (**B**) Infiltrated leukocytes were isolated from spinal cord and brain. Viable CD45^+^CD4^+^ or CD45^+^CD19^+^ cell numbers from spinal cord and brain were determined by FACS analysis. (**C**,**D**) The cells were subjected to intracellular cytokine staining to detect percentage of cytokine expression in CD4^+^ T cell (**C**) and CD19^+^ B cell compartments (**D**). (**E**) Draining LN cells were isolated and counted, and analyzed for cell surface expression of CD4 or CD19 by FACS. (**F**) The draining LN cells were stimulated with MOG_35-55_-peptide for 48 h and subjected to ^3^H-thymidine incorporation assay. Data are presented as mean CPM ± S.E.M. of responses of 4 replicate cultures. Data represents > 3 independent experiments (**P < 0.01; ***P < 0.001; ****P < 0.0001).

Experimental autoimmune encephalomyelitis (EAE) and EAU share essential immuno-pathogenic features characterized by progressive relapsing–remitting inflammation induced by the recruitment Th17 and/or Th1 into the CNS. We therefore used the CD19-STAT3 KO to evaluate whether STAT3 deletion in B cells enhances inflammation in the EAE model. We induced EAE in C57BL/6J mice by immunization with MOG_35-55_-peptide in CFA. While the CD19-STAT3KO mice developed severe EAE characterized by infiltration of inflammatory cells into the brain/spinal cord, development of flaccid tail, paraparesis, front/hind limb paralysis and moribund state, these hallmark features of EAE were significantly reduced in WT control mice (Fig. 7A). We isolated CD45^+^ leucocytes that infiltrated the spinal cord and brain of the EAE mice and show here that the number of CD4^+^ and CD19^+^ leucocytes were significantly higher in CD19-STAT3KO compared to WT mice (Fig. 7B). Intracellular cytokine staining analysis further show significantly higher percentage of Th1 and Th17 cells (Fig. 7C) while the frequency of IL-10- and IL-35 Breg cells in these tissues of the CD19-STAT3KO EAE mice was significantly lower compared to WT (Fig. 7D). Furthermore, the percentages of Ki-67^+^ cells correlated with increase of CD19^+^ lymphocytes in the draining LN (Fig. 7E) and MOG-specific encephalitogenic CD19-STAT3KO B cells in the draining LN proliferate faster than WT mice with EAE (Fig. 7F). Collectively, these observations in CD19-STAT3KO mice with EAE recapitulate essential immunopathogenic features we observed in EAU model.

## Discussion

STAT3 mediates cell survival, proliferation and inflammation and global deletion of *stat3* is embryonic lethal with the mice dying by 3 weeks of age. Thus, study of intrinsic function of STAT3 in any cell type has relied on selective deletion of *stat3* by Cre/Lox recombineering technology. In B cells, STAT3 positively regulates an early step in B-cell development important for germinal center maintenance^23,37,43^. In this study, we investigated whether loss of STAT3 in mature B cells would promote resistance or susceptibility to uveitis and to evaluate the safety and efficacy of targeting STAT3 pathways as immunotherapy for this potentially blinding disease.

We show that STAT3 regulates B cell proliferation through upregulation of lymphocyte quiescence factors such as Foxo1 and Foxo3a, contributes to the maintenance of homeostatic levels of B cells and regulates glycolytic activities required to meet metabolic and bioenergetic demands of B cells. We also reveal a complex role of STAT3 in regulating cycle events in B cells and B cell activation. In addition, we provide experimental evidence that expression of STAT3 in CD19 B cells confers protection from CNS autoimmune diseases as evidenced by exacerbation of uveitis in CD19-STAT3KO mice. In contrast to mild uveitis observed in WT mice, mice with conditional deletion of STAT3 in the B cell compartment developed severe EAU characterized by massive infiltration of inflammatory cells into the retina and targeted destruction of photoreceptor cells. Furthermore, light-adapted or dark-adapted ERG analysis revealed significantly lower a-wave and b-wave amplitudes in eyes of CD19-STAT3KO mice, suggesting significant decline of cone and rod signaling functions and visual impairment in the CD19 STAT3KO mice. Indeed, across the board all hallmarks of severe acute uveitis were observed in CD19-STAT3KO mice and disease severity correlated with increase of Th17 cells that secrete IL-17 and IFN-γ. Moreover, expression of costimulatory molecules was markedly upregulated on CD19-STAT3KO B cells, suggesting that STAT3 attenuates expression of costimulatory molecules on B cells and might thereby serve to restrain excessive activation of T cells that can induce autoimmunity. It is also of note that the expansion of uveitogenic T cells and exacerbated disease in CD19-STAT3KO mice correlated with the reduction of cell surface expression of Lag3 and diminished capacity to suppress T cell effector functions or induce T cell exhaustion. EAE and EAU share essential immunopathogenic features of human uveitis and multiple sclerosis respectively. These mouse CNS autoimmune diseases are characterized by progressive relapsing–remitting inflammation induced by Th17 and/or Th1 cells and they provide a platform for developing and evaluating effective therapies for uveitis or multiple sclerosis. We show here that STAT3 deletion in B cells correlated with enhanced inflammation in the brain and spinal cord and correlated with severe encephalomyelitis. Recapitulating essential immunopathogenic features of EAU in the EAE model thus support our conclusion that STAT3 deletion in B cells enhances neuroinflammation and that the effects observed in EAU may be applicable to other neuroinflammatory diseases. It is of note that previous reports have used mice with conditionally deleted Stat3 in the B-cell lineage (Stat3^fl/fl^CD19^Cre/+^) to establish the role of STAT3 in early stages of B-cell development^37^. However, limited studies have examined intrinsic functions of Stat3 in mature B cells and those reports show that Stat3 plays a role in T-dependent terminal differentiation of IgG B cells and indicated that the requirement for Stat3 in B cells is limited to plasma cell differentiation or as negative regulator controlling IgE class switching^23,44^. Data presented in our study provide new and important insight into the intrinsic role of Stat3 pathway of mature B cells by showing that loss of STAT3 in B cells exacerbates uveitis or encephalomyelitis and underscore the role of Stat3 pathway of B cells in peripheral immune tolerance and regulation of autoimmune disease.

Although it is well-established that IL-10 producing Tregs suppress inflammation and organ-specific autoimmune diseases, recent studies have identified regulatory B cells (Bregs) that secrete IL-10 and/or IL-35 as critical regulators of immunity during autoimmune diseases. These Bregs also regulate inflammation through upregulation of inhibitory receptors including Pd-1 or Lag3 and attenuate functions of proinflammatory T cells through induction of T cell exhaustion^45^. In this study, we found that severe uveitis in the CD19-STAT3KO mouse correlated with marked reduction of IL-10-producing Tregs and Bregs, as well as, IL-35-producing Tregs and Bregs. Moreover, the level of Lag3 in these Bregs are markedly reduced in CD19-STAT3KO B cells consistent with the exacerbated EAU in the CD19-STAT3KO mice. Although previous reports have highlighted the requirement of STAT3 pathways for the inhibitory functions of Tregs and Bregs^25^, data presented here suggest that STAT3 may also play a role in regulating the differentiation or expansion of Treg and Bregs. Interestingly, the decrease in Treg and Breg subsets and marked increase in Th17 signature cytokines during EAU and EAE in CD19-STAT3KO mice may provide a mechanistic link between loss of STAT3 pathways in B cells and development of severe uveitis. In this context, we have demonstrated that loss of STAT3 in B cells coincided with significant increase in cell surface expression of CD80 and CD86 (Fig. 6A,B), suggesting that CD19-STAT3KO B cells might exhibit increased capacity to serve as antigen-presenting cells. In fact, the capacity to activate T cells in situ, at target site of inflammation (e.g. the neuroretina or brain), plays important role in perpetuating pathology during neuroinflammation. Data presented here thus suggest that increased pathology during EAU or EAE can be attributed to enhanced activation of pathogenic CD4^+^ T cells in retina or brain by CD19-STAT3KO B cells and diminished capacity of regulatory cells to suppress expansion of Th17 could contribute to enhanced ocular pathology.

Uveitis comprises a heterogeneous group of potentially sight-threatening inflammatory diseases that includes sympathetic ophthalmia, birdshot retinochoroidopathy, Behcet’s disease, Vogt-Koyanagi–Harada disease, and ocular sarcoidosis and accounts for 10% of severe visual handicaps in the United States^46,47^. Conventional treatments of uveitis such as corticosteroids can cause serious systemic side effects, and there is considerable impetus to seek alternative therapies such as biologics that can be used to target proinflammatory pathways that mediate autoimmune diseases. Previous reports have shown that STAT3 is required for the development of pathogenic Th17 cells that mediate uveitis and mice with targeted deletion of STAT3 in T cells do not develop uveitis^20^. This has led to the suggestion that STAT3 inhibitors can be used to suppress or modulate uveitis and other CNS autoimmune diseases mediated by Th17 cells. However, data presented in this study shows that loss of STAT3 in B cells exacerbates uveitis by inducing excessive expansion of Th17 cells and suppressing Breg cells, suggesting that STAT3 signaling pathway is essential for immune regulatory functions of B cells and that augmenting STAT3 pathway in B cells be used to suppress uveitis. However, Thus, targeting STAT3 pathway in lymphocytes may produce unpredictable outcome as indicated by divergent effects exhibited dependent on the immune cell type. In addition, neurons and photoreceptors in the brain or neuroretina constantly interact with neurotrophic cytokines and growth factors such as ciliary neurotrophic factor (CNTF), oncostatin M (OSM) and leukemia and inhibitory factor (LIF) that activate STAT3^48^. Activation of STAT3 pathway by these cytokines has been shown to exert neuroprotective functions in the retina leading to proposal of STAT3 augmentation therapy for retinal dystrophies^49,50^. On the other hand, persistent activation of STAT3 pathway in the retina has also been shown to induce vision impairment and retinal degenerative changes in ageing mice, further underscoring unpredictable effects of targeting STAT3 pathway^51^. Taken together, data suggest that much caution should be exercised in efforts to modulate STAT3 pathways as therapy for inflammatory diseases as its role in inducing differentiation of pathogenic Th17 cells has to be weighed against its role in promoting cell survival and in suppressing proinflammatory costimulatory molecules or inhibitory receptors that restrain exuberant T cell activities that can cause autoimmunity.

## Supplementary information

Supplementary Figure S1.

Supplementary Figure S2.

Supplementary Figure S3.
