# Supplementary figures and images for: STAT3 deficiency in B cells exacerbates uveitis by promoting expansion of pathogenic lymphocytes and suppressing regulatory B cells (Bregs) and Tregs
Source: Sci Rep. 2020 Oct 1;10:16188. doi: 10.1038/s41598-020-73093-1 (PMC7529787; doi:10.1038/s41598-020-73093-1)

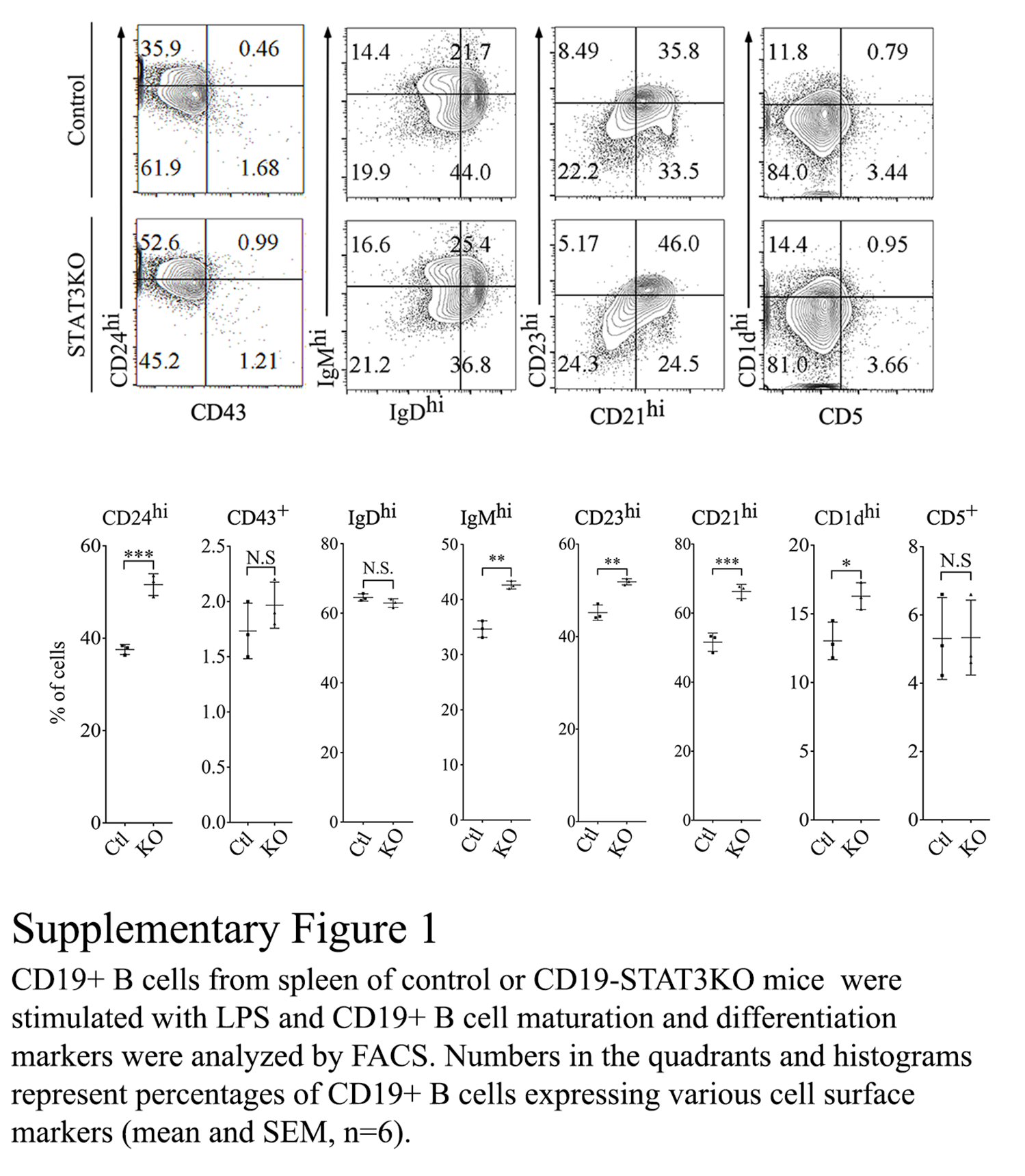

Supplement: Supplementary file 1 — Supplementary Figure S1. [file 41598_2020_73093_MOESM1_ESM.tif]

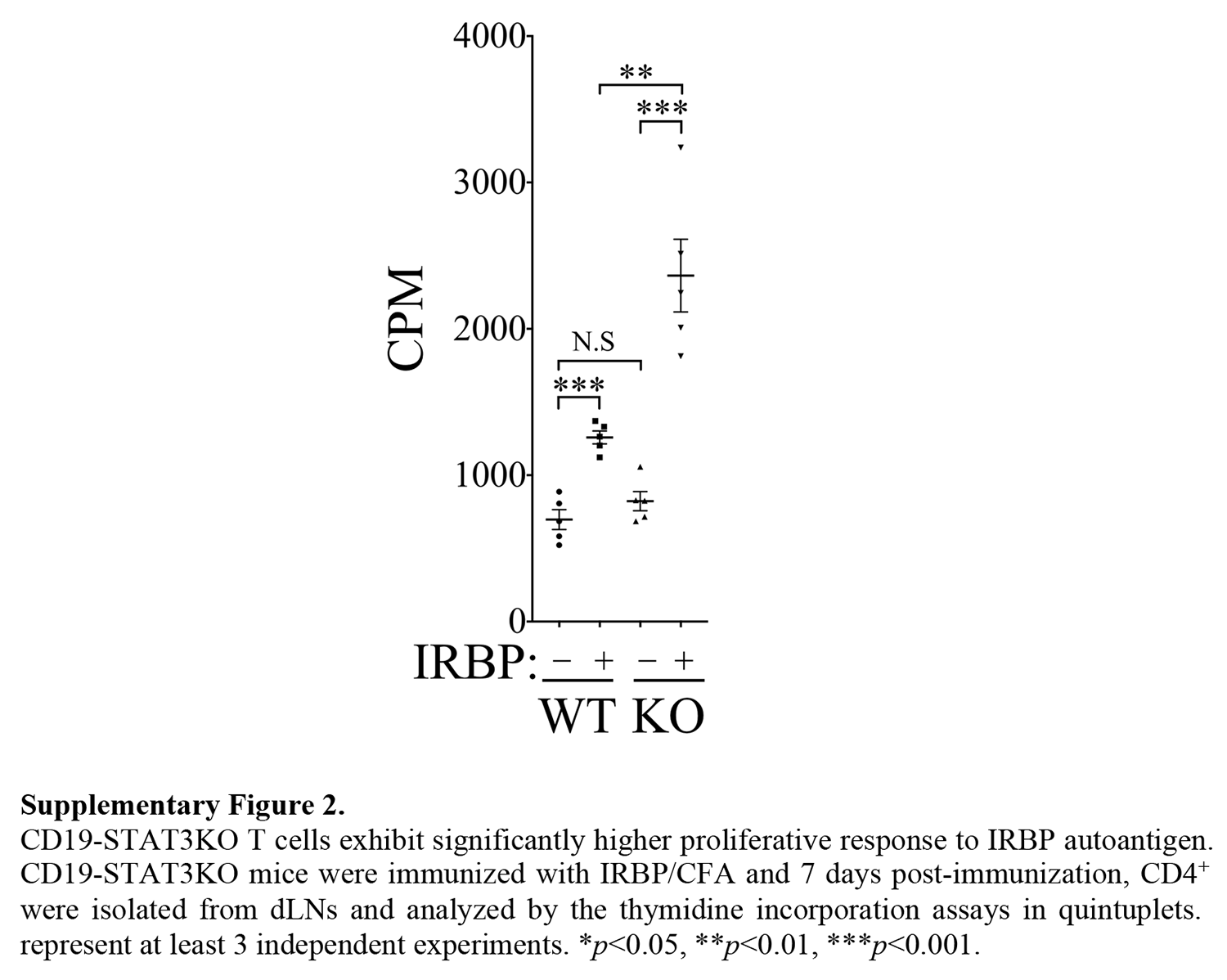

Supplement: Supplementary file 2 — Supplementary Figure S2. [file 41598_2020_73093_MOESM2_ESM.tif]

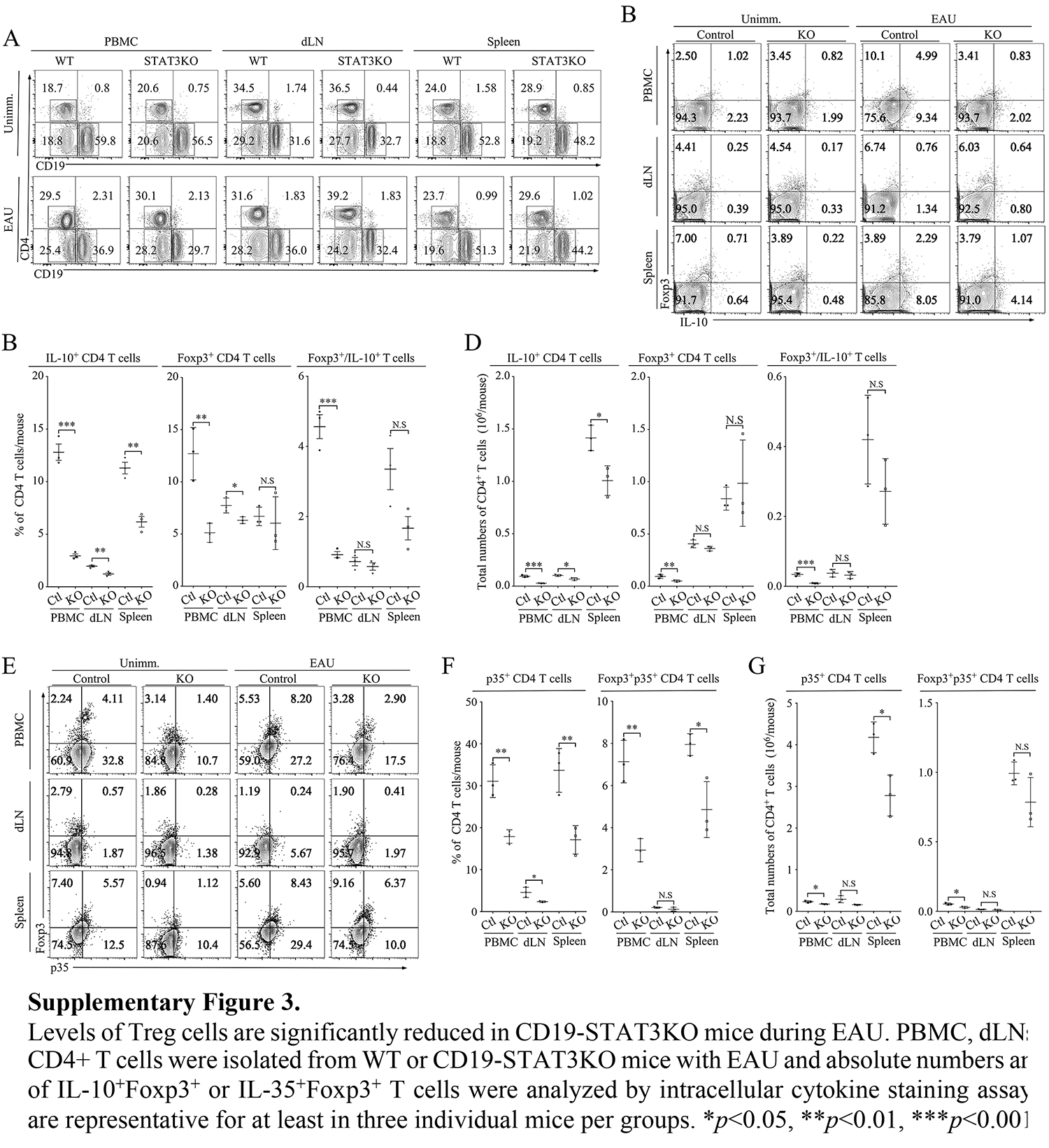

Supplement: Supplementary file 3 — Supplementary Figure S3. [file 41598_2020_73093_MOESM3_ESM.tif]
